# Effect and associated factors of a clinical pharmacy model in the incidence of medication errors (EACPharModel) in the Hospital Pablo Tobón Uribe: study protocol for a stepped wedge randomized controlled trial (NCT03338725)

**DOI:** 10.1186/s13063-019-3945-8

**Published:** 2020-01-06

**Authors:** J. Granados, A. Salazar-Ospina, J. P. Botero-Aguirre, A. F. Valencia-Quintero, N. Ortiz, P. Amariles

**Affiliations:** 1 Grupo Promoción y Prevención Farmacéutica, Facultad de Ciencias Farmacéuticas y Alimentarias, Universidad de Antioquia, Calle 70 No 52-21, Medellín, Colombia; 2 Grupo de investigación en Tecnología en Regencia de Farmacia, Universidad de Antioquia U de A, Calle 70 No 52-21, Medellín, Colombia; 3 Grupo Investigación clínica HPTU., Hospital Pablo Tobón Uribe, Calle 78B #69-240, Medellín, Antioquia 05001000 Colombia

**Keywords:** Medication errors, Drug-related problems, Pharmacy service, Hospital (clinical pharmacy services), Pharmacists

## Abstract

**Background:**

According to WHO, medication error (ME) is a subject that requires attention at all levels of care to reduce severe and preventable damage related to medication use. Clinical pharmacy practice standards have been proposed around the world so that the pharmacist, as part of a multidisciplinary health team, can help improve patient safety; however, further evidence derived from adequate studies is needed to demonstrate this. This study aims to assess the effect of a clinical pharmacy practice model (CPPM) in preventing MEs associated with the medication use process.

**Methods:**

A prospective, stepped-wedge, cluster-randomized, controlled trial with a duration of 14 months will be performed to compare the effect of a CPPM along with the usual care process of patients in the Pablo Tobón Uribe Hospital (Medellin, Colombia). The study is designed as a cluster-randomized controlled trial, involving five hospital wards (clusters) and 720 patients. Medical wards are allocated to interventions using a stepped-wedge design. Clusters are initially assigned to the control group. After a 2-month observation period, hospital clusters were randomly allocated to the intervention group. Study outcomes will be assessed at baseline and at 2, 4, 6, 8, 10, and 12 months after randomization. The primary outcome will be to assess the effect of a CPPM on the incidence of medication errors associated with the medication use process. Drug-related problems and factors that contribute to the occurrence of MEs will be assessed as secondary outcomes. Statistical analyses will be performed using a mixed model, with the treatment group and time as fixed effects and the clustering structure as a random effect. Statistical analysis will be performed using Pearson chi-square tests and Student’s t-tests, and a *P* value < 0.05 will be considered statistically significant.

**Discussion:**

As far as we know, this is the first stepped-wedge, cluster-randomized, controlled trial designed to assess the change of a CPPM on the incidence of medication errors in a hospital in Colombia, and it could generate valuable information about a standardized and patient-centered clinical pharmacy model to improve the safety of inpatient care.

**Trial registration:**

ClinicalTrials.gov, NCT03338725. Registered on 9 November 2017. The first patient was randomized on 2 February 2018.

**Protocol version:**

0010112018JG

## Background

The report published by the United States Institute of Medicine entitled “To err is human, building a safer health system” identified the serious problem with medical errors in the healthcare system. Medical error represents a severe public health problem that may have implications for patient safety and may contribute to a considerable increase in the cost of health care [1]. The report also indicated that during hospital care, approximately 4% of hospitalized patients are at risk of suffering some damage caused by health care errors or adverse events, of which 70% may generate temporary disability and 14% fatal incidents, causing between 44,000 and 98,000 deaths each year [1, 2]. For the year 2013, the annual mortality due to avoidable errors in health care in the United States was estimated as being at least 251,000 deaths [3].

Approximately 37% of adverse events that occur during the health care process are related to medication errors (ME). Medication errors in a hospital may represent between 8.0 and 19.6% of drug administrations, particularly during the prescription and medication administration process [4]. According to the National Coordinating Council of Medication Error Reporting and Prevention (NCC MERP), a medication error is defined as “any preventable event that may cause or lead to inappropriate medication use or patient harm while the medication is under the control of the health care professional, patient, or consumer.” These errors may be associated with any phase of the drug delivery process, from prescription to drug administration, at any place where medications are administered [5].

The World Health Organization (WHO) in the last decade has focused its efforts on patient safety, implementing a series of actions aimed at reducing adverse events related to health care [6, 7] as well as correcting deficiencies in different health systems that may lead to medication errors and severe health damage. According to these initiatives and based on safety reports related to the incidence of medication errors, different strategies have been implemented as a global initiative to reduce medication errors, including, for example, the implementation of clinical pharmacy services, where the pharmacist plays a fundamental role in the healthcare team as a support professional in the optimization of pharmacotherapy [8] as well as in the active search for medication errors and the promotion of pharmacological safety strategies. These strategies help to prevent unnecessary associated injuries during the therapeutic use of medications [9–14]. Some studies suggest that pharmacists make no significant contribution to the reduction in MEs, but they can reduce preventable adverse drug events. However, a need exists for high-quality studies to explore the effect of the pharmacists on patient safety [15].

In mid-2016, the Pablo Tobón Uribe Hospital (PTUH), a highly complex university institution in the city of Medellin (Colombia), standardized a clinical pharmacy practice model that incorporated the activities of the pharmacist into the multidisciplinary health team to provide clinical pharmacy services [16] and to improve patient safety. Currently, this model is actively applied in the hospital; however, knowledge of the full effect of the model on patient safety, as framed in the WHO initiative to reduce medication errors, is needed [17]. The EACPharModel study aims to assess the effectiveness of a clinical pharmacy practice model in reducing the incidence of medication errors in a tertiary university hospital.

## Methods/Design

### Design

A 14-month, randomized, controlled, prospective, single-center, stepped-wedge, clinical trial will be performed to compare the effect of a CPPM in the incidence of medication errors.

A stepped-wedge cluster-randomized trial is proposed because the randomization of patients is not possible and because of ethical issues. A randomized cluster trial would be difficult to conduct because the cluster number would be high, and half of the clusters would not benefit from the intervention [18, 19].

**Fig. 1:**
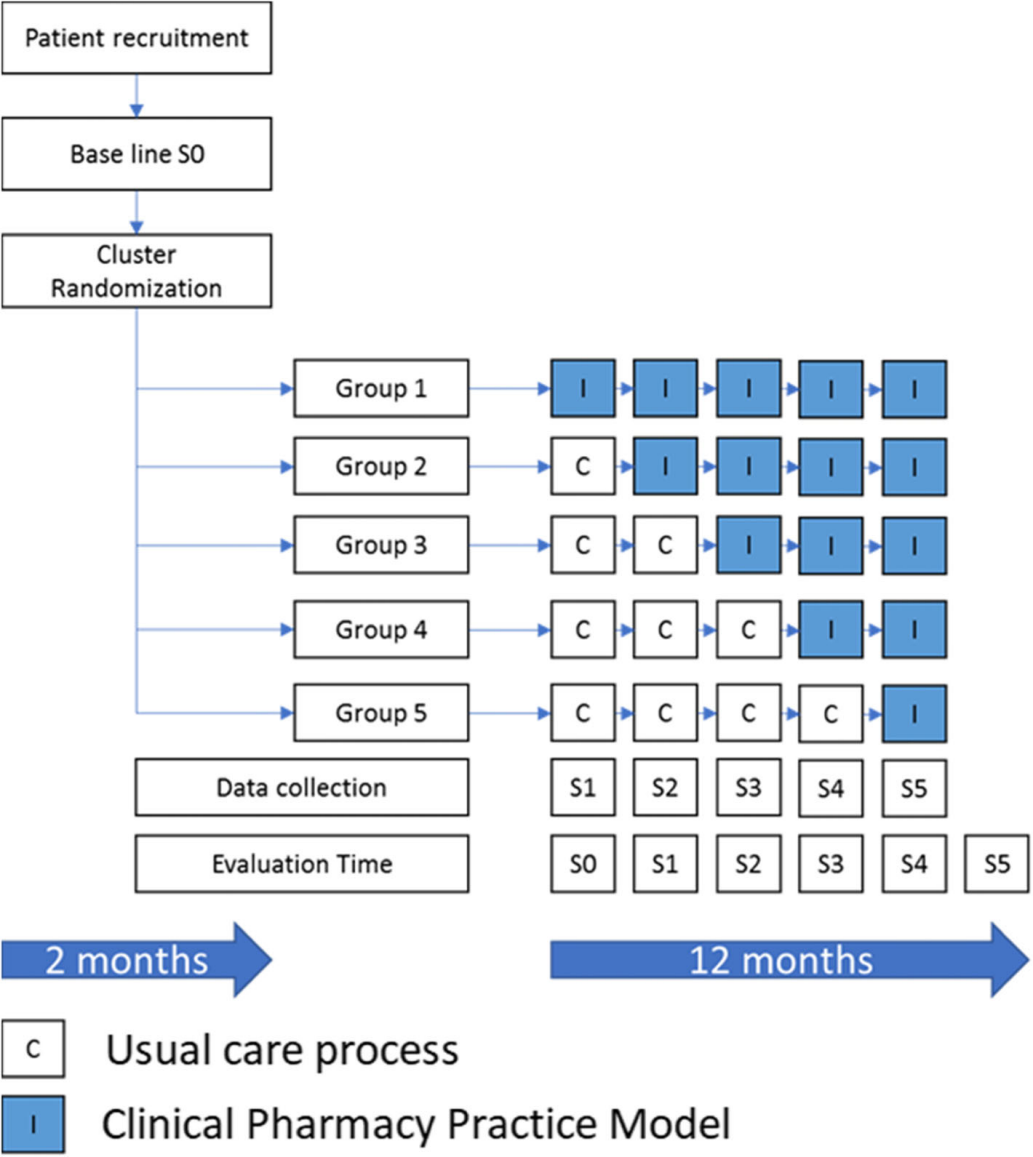
Design and timeframe of the EACPharModel study. The outcomes to be assessed during each evaluation stage are the incidence of medication error, time to the error discovery, and time to error recovery

A stepped-wedge randomized trial designs involves the sequential roll-out of an intervention to participants over several periods, and by the end of the study, all participants will have received the intervention, although the order in which participants receive the intervention is determined at random. The clusters will be hospital wards. Each hospital cluster will start with a control period (Baseline S0) and switch to an interventional period after randomization. The study design will be composed of six consecutive 60-day periods (Fig. 1). During each 60-day period, one further hospital cluster will switch to the interventional period, until all the clusters are in the interventional period during the final 60-day period. The randomization by the trial methodologist will define the point at which each hospital cluster will switch from the control to the interventional periods (Table 1).

**Table 1:** Clusters, participants in the control period, and switching period

Hospital cluster (Hospital ID)	Main specialty	Participants in the control period	Intervention starting period
B-08-1	Internal medicine and surgery	Pharmacy technicians	Period 1
B-05-2	Internal medicine	Pharmacy technicians	Period 2
B-03-1	Orthopedics	Pharmacy technicians	Period 3
B-08-2	Internal medicine	Pharmacy technicians	Period 4
B-07-2	Internal medicine	Pharmacy technicians	Period 5

### Setting

The study is to be conducted with hospitalized patients who presented at the Pablo Tobón Uribe Hospital and were prescribed five or more drugs during their hospital stay. PTUH is a tertiary care university hospital with 483 beds (395 adults and 88 pediatrics). Adult beds are distributed in 17 hospital wards and three intensive care units (ICU); however, for this study, five hospital wards will be used, including mainly the internal medicine and orthopedic/infectious disease specialties because these wards have the largest number of patients.

### Study population

The study will recruit 720 patients, and a pharmacist will evaluate whether each patient meets all the inclusion criteria.

The inclusion criteria will be the following:

- Patients at least 18 years old
- Hospitalized patients in the Pablo Tobón Uribe Hospital
- Patients receive at least five drugs in their pharmacological therapy

The exclusion criterion is a ward stay of less than 24 h.

Withdrawal criteria include the following:

- Protocol deviation and violation
- Decrease of drug prescriptions to four or fewer during the hospital stay

### Patient recruitment and group assignment

A pharmacist is responsible for the recruitment of potential patients to the hospitalization wards. A hospitalization ward will be defined as a cluster, and 2 months are considered a step. For step 0 or baseline, no cluster will have the intervention; for step 1, a cluster will be randomly selected to receive the intervention and continue until the end of the study; and at step 2, another cluster will be randomly selected to receive the intervention and continue until the end of the study; this will continue until step 5, when all clusters will have the intervention and the inclusion of the participants within the cluster will be dynamic.

**Fig. 2:**
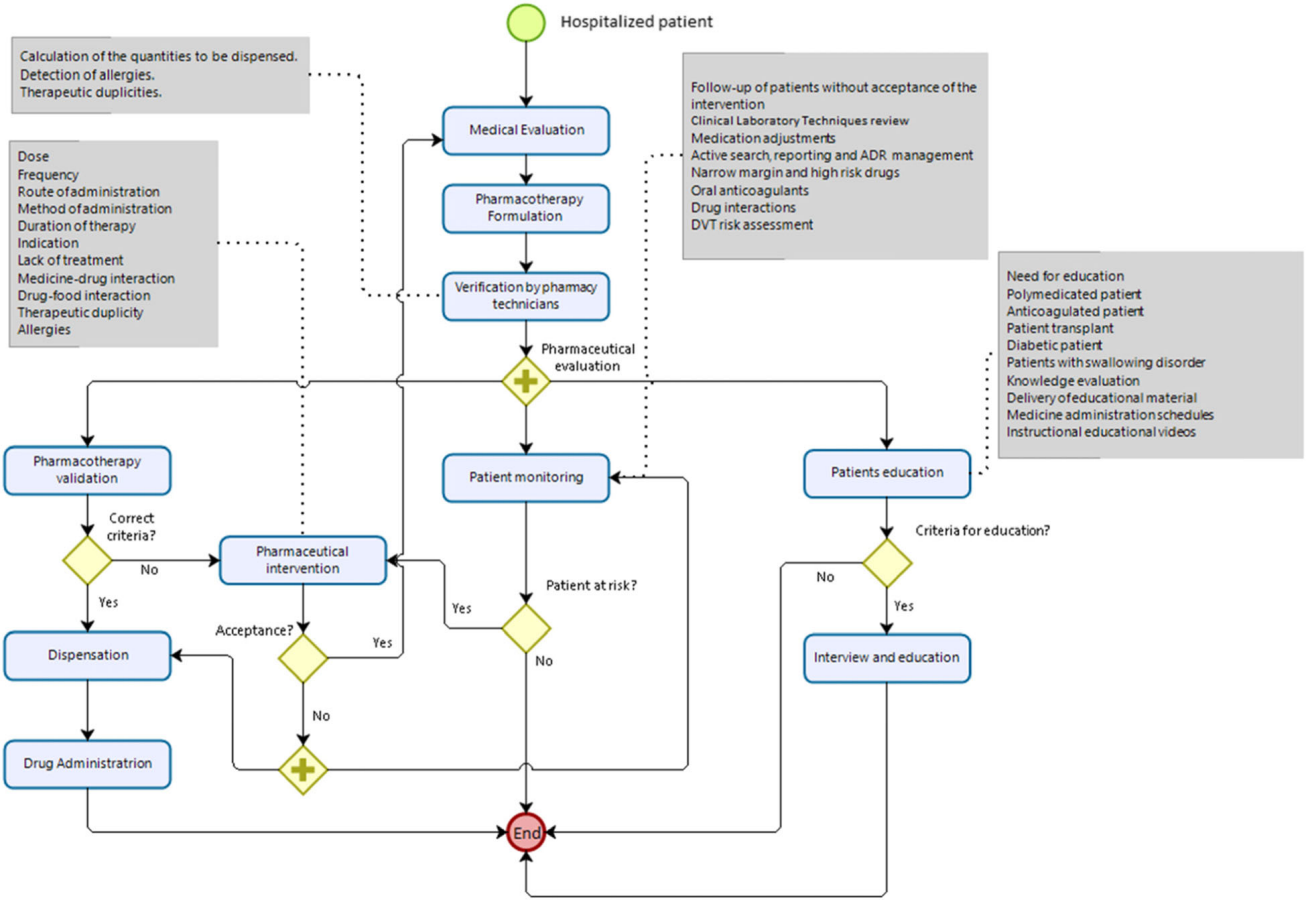
Clinical pharmacy practice model

The study procedures and assessments are outlined in the Standard Protocol Items: Recommendations for Interventional Trials (SPIRIT) and are shown in Fig. 2.

### Outcomes

The primary outcome to be measured will assess whether the clinical pharmacy model can change the incidence of medication errors. Among the secondary outcomes are the following: 1) the quantification and classification of medication errors, 2) identification of the factors that contribute to the occurrence of medication errors, and 3) estimation of the probability that a subject remains without medication errors and the measurement of the time until medication errors will resolve.

### Intervention design

#### Intervention group

Attention under the clinical pharmacy practice model of the hospital is focused on the following activities: (1) participation in medical rounds (at least 3 days per week); (2) therapeutic monitoring, which consists of reviewing patients’ medical records and providing verbal or written follow-up concerning their clinical conditions (involves repeated monitoring, with review of all medication orders and documentation of pharmaceutical interventions); (3) identification and management of adverse drug reactions (ADR), which consists of detecting potential ADR, providing and documenting appropriate follow-up until the ADR has resolved, and reporting ADRs to the national pharmacovigilance program; (4) pharmacological counselling to patients, which involves providing information about the proper use of medications to patients and/or family members during the hospital stay or after discharge; (5) pharmacotherapy validation, in which pharmacists conduct an appropriate review of medical orders and determine, for example, the correct dosage, correct frequency, correct route of administration, correct administration, correct duration of therapy, indicated drugs, contraindicated drugs, lack of treatment, drug-drug interactions, drug-food interactions, therapeutic duplicity, and allergies.

**Fig. 3:**
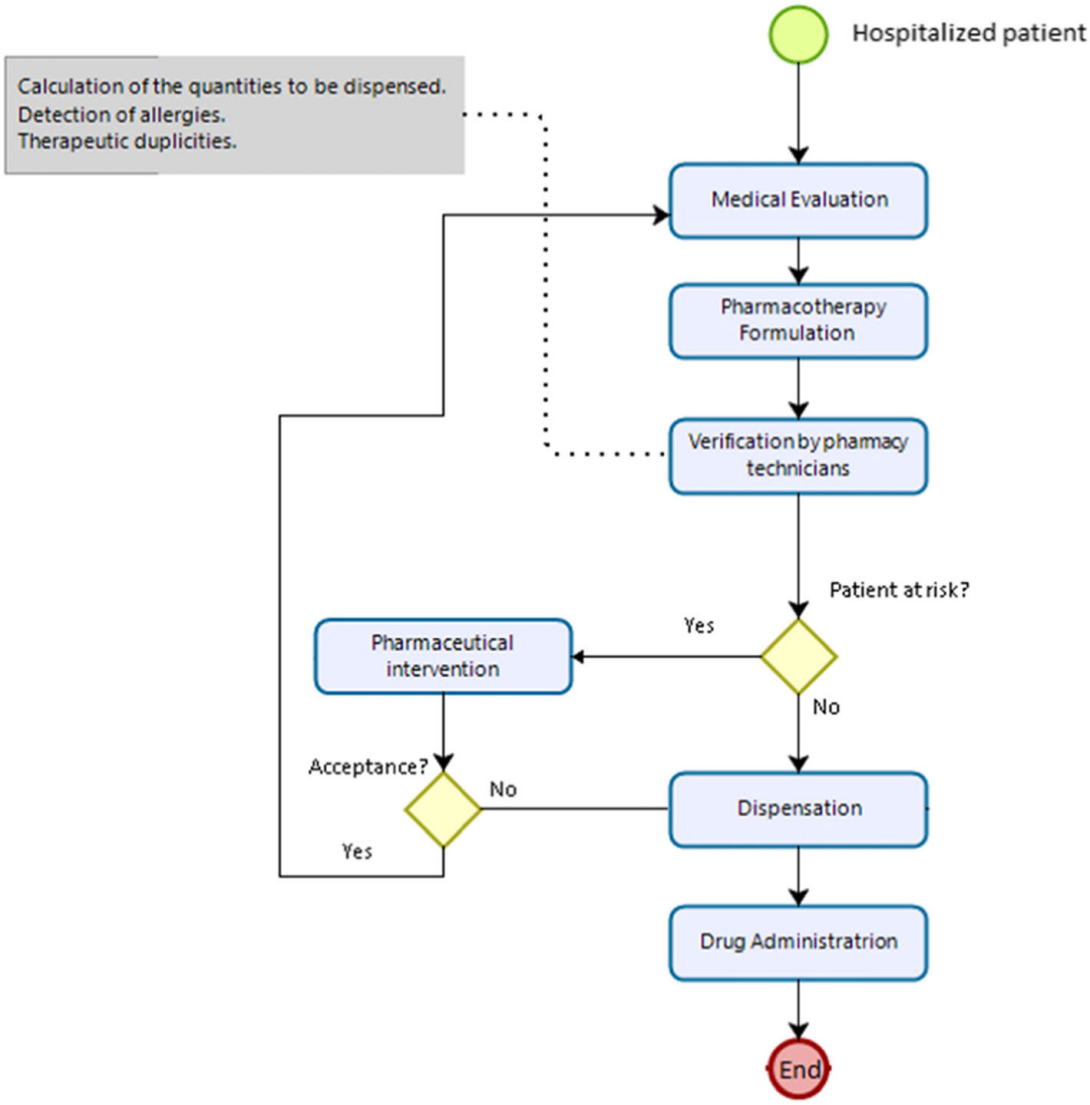
Usual care process

Every day, the pharmacist in the morning will provide a list of patients that they must evaluate as specified in activities 2, 3, and 5 or including other patients when necessary or when any patient requires attention. The model is shown in Fig. 3.

#### Control group

**Fig. 4:**
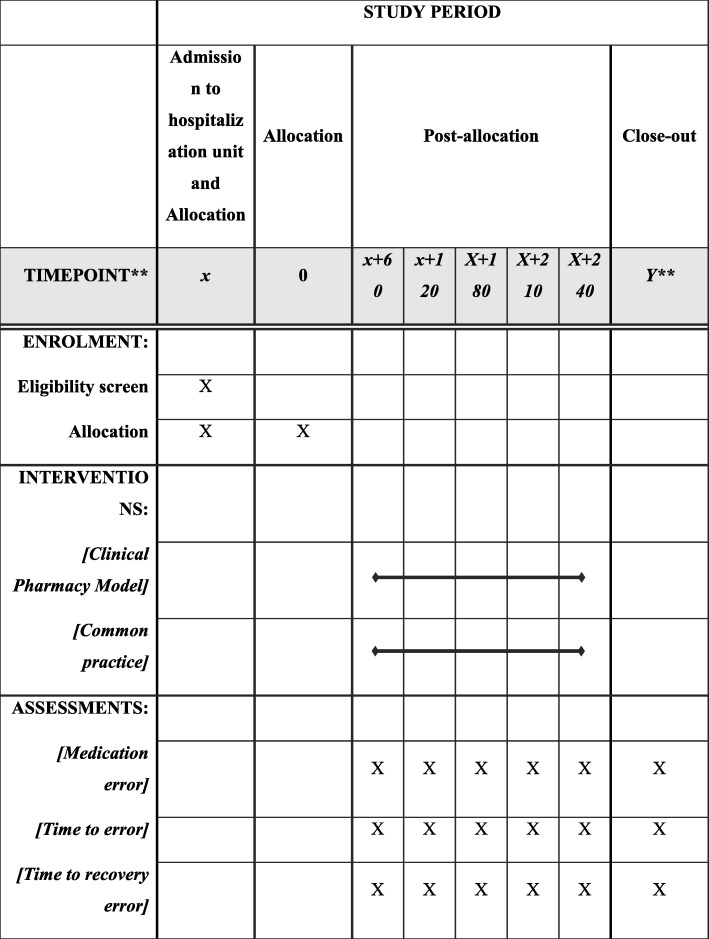
Standard protocol items: recommendation for interventional trials (SPIRIT) figure. * X is 1–60 days (baseline). ** Y is a period after the end of the post-discharge follow-up

The usual patient care process includes a medical evaluation and the respective formulation of pharmacotherapy (Fig. 4). Later, the pharmacy technicians will use a spreadsheet to verify the quantities that will be dispensed, allergy detection, therapeutic duplicities, and finally, the medicine will be dispensed.

### Limitations and potential bias

As the collection of the information will be based on the review of medical records, some sub-registries may be present that will affect medication error detection. However, PTUH is an institution accredited by the Joint Commission International, which has made it possible to adequately standardize the process, including the quality of the medical records, which is therefore expected to control this bias.

### Randomization

Clusters will be assigned to the intervention group or the control group through a computer-generated randomization sequence, designed by a person external to the study and otherwise unrelated to it, using Microsoft Excel® (version 2010, Microsoft® Corporation, Redmond, Wash.).

### Blinding

An external evaluation group made up of an internist and a pharmacist will be established to evaluate whether a medication error occurred and whether it resolved. This evaluation will be done once the patient’s hospitalization has ended and the patient discharged. Additionally, the evaluators will be blinded and will not know if the patients were in the clinical pharmacy model; the notes of the pharmacist in the medical record will not be available.

### Sample size calculation

For the sample size calculation, we use the package SWSamp [20] in R software version 3.2. The overall incidence of medication errors of 15% was a reference value [21]. The calculation of the sample size was made by accepting an alpha error of 0.05, a beta error of 0.84, five clusters, six steps, two months for each step, 24 participants per cluster, and a coefficient of variation (k) of 0.15. The number of individuals needed to detect a difference equal to or more significant than 10% was 720.

### Data collection

Data will be collected from February 2018 to January 2019. The study will extend over 14 months, the recruitment period will be 12 months, and patients will be evaluated for 2 months, starting from the date of their recruitment. Once the enrolment period ends, the final 2 months will be used only to evaluate the latest patients. The data obtained in this study will register in an electronic database. Data regarding the medication history, interviews with the pharmacist, health status, plans of action, as well as the data related to the primary outcome, will be registered.

A specially trained clinical pharmacist and an internist will make up the clinical staff that will accurately measure the medical record of each patient at baseline (t0), 2 months post-baseline (t1), 4 months post-baseline (t2), 6 months post-baseline (t3), 8 months post-baseline (t4), and 10 months post-baseline (t5). The clinical staff will be an external evaluation group that will evaluate if a medication error has occurred and whether it resolves. This evaluation will be done once the patient’s hospitalization has ended and the patient discharged.

### Data management

Only the pharmacist and the physician involved in the trial will have access to the data entry software. The data will be controlled and formatted to prevent errors in transcription. Data will be stored in a database PostgreSQL and accessed with software developed in VB.net specifically for the trial. The database includes information about medications and treatments, physical assessment records, laboratory tests, and hospitalizations. The database shows reports of a patient’s assessment forms, allows monitoring of data quality, and generates reports according to the user’s needs. Researchers involved in this study will be the only ones with access to the information. We will back up the database daily in two different computers to protect the information. Table 2 shows the personnel involved in the study and their function.

**Table 2:** Function of researchers

Person	Function	Blinding
Pharmacist and epidemiology 1	Database completion	No
Pharmacist and epidemiology 2	Recruitment Database completion	No
Pharmacist and statistician	Recruitment Statistical analysis. Database completion	No
Pharmacist	Evaluation of medical records outcome evaluation	Yes
Physician	Evaluation of medical records outcome evaluation	Yes

### Missing data

We will inspect missing data, and if less than 5% is missing, we will use imputation by the median values for the continuous variables and mode for the categorical ones.

### Statistical analysis

The statistical analyses of the full-analysis set will follow the intention-to-treat (ITT) principle with R software version 3.2, using the patient-related observations from the medical records as the unit of analysis, a dataset that includes all subjects in the assigned cluster and meets all inclusion criteria. Baseline and demographic characteristics will be analyzed descriptively (number of valid cases, mean, standard deviation, median, interquartile range, and proportions for qualitative variables). A mixed model will evaluate the primary hypothesis with the treatment group and time as the fixed effects and the clustering structure as a random effect. A significance level set to alpha = 5% (two-sided) will be used to compare proportions. Comparisons for categorical variables will be conducted using the chi-square test (or the Fisher exact test when appropriate) and for continuous variables by using the independent-sample t-test (if the distributions are not typical, as determined by the Kolmogorov-Smirnov test, we will use the Mann-Whitney U test). Student’s t-test (between study groups) will be used to compare means, and the odds ratios (ORs) and 95% confidence intervals (CIs) will be estimated as well. The mean number and duration of relapse events, normalized to person-time, will be compared between groups using Student’s t-test. Multivariate analyses will be performed to explain the association of multiple variables with the factors significantly related to primary outcome. The sociodemographic and clinical variables to be assessed are sex, age, social security system, scholarship, weight, height, allergies, having a caregiver, diagnosis of admission, hospitalization in the previous 6 months, number of services, previous stay in intensive care unit, adverse drug reaction, colonized patient, hospital stay length, and number of medications. Table 3 shows the operationalization of variables, and Table 4 show statistical analysis for outcome.

**Table 3:** Operationalization of variables

Variable	Operational definition	Nature	Unit of measurement (Categorization)
Sex	Biological condition at birth	Nominal qualitative	1. Man 2. Woman
Age	Age in years	Quantitative	Age in years
Social security system	Membership social security system	Nominal qualitative	1. Contributory 2. Subsidized
Scholarship	Schooling	Qualitative ordinal	1. No studies 2. Primary 3. High School 4. College undergraduate 5. University postgraduate degree
Weight	Patient weight	Quantitative	Weight in Kg
Height	Patient height	Quantitative	Height in cm
Allergies	Allergic antecedents	Nominal qualitative	1. Yes 2. No
To have caregiver	Family or friend who accompanies during hospitalization	Nominal qualitative	1. Yes 2. No
Diagnosis of admission	Admission diagnosis	Nominal qualitative	–
Hospitalization 6 months before	Hospitalization 6 months before	Nominal qualitative	1. Yes 2. No
Number of services received	Specialties that are treating the patient	Quantitative	Count
Previous stay in intensive care unit	Previous stay in intensive care unit	Nominal qualitative	1. Yes 2. No
Adverse drug reaction	Adverse drug reaction	Nominal qualitative	1. Yes 2. No
Colonized patient	Patient isolated unit because a multi-resistant bacterium colonizes him	Nominal qualitative	1. Yes 2. No
Hospital stay length	Hospitalization days	Quantitative	Count
Number of medications	Number of drugs prescribed	Quantitative	Count

**Table 4:** Statistical analysis for outcome

Type Analysis	Measure
Univariate analysis	Population characterization
Bi-varied analysis	Estimation of the association (RR) between the clinical pharmacy model and the incidence of medication errors.
	The difference of means in the hospital stay length
Multivariate analysis	Poisson regression to determine the variables that most influence the MEs.
Survival analysis	The difference in time in the presence of a medication error.
	The difference in time in the resolution of a medication error.

## Discussion

Medication errors account for approximately 37% of adverse events that occur during health care [4]. The harm caused by medication errors in poly-medicated patients is frequent, constituting a potential cause of death and increasing the costs associated with health [22]. In the last decade, WHO has focused its efforts on implementing actions aimed at reducing the adverse events related to health care as well as correcting deficiencies that may lead to medication errors and severe damage [23]. Different studies have tried interventions to reduce these errors, which include the development of computerized physician order entry (CPOE), use of clinical decision support systems, standardization of protocols for the treatment and management of medication in different health problems, and the implementation of education and training programs for health staff, as well as the incorporation of pharmacists into the patient care team, thereby allowing activities that improve medication safety [24–26]. In another stepped-wedge study, the MEDREV Working Group et al. sought to measure the impact of collaborative pharmaceutical care on preventable medication error rates, but their results are not yet available [27].

Worldwide, different clinical pharmacy models have described multiple strategies from the pharmaceutical view for improving safety inpatient care; however, we did not find results where they evaluated the change in the incidence of medication errors. For that, Pablo Tobón Uribe Hospital, framed under international standards of safety, quality, and care (Joint Commission International), has standardized a clinical pharmacy model contributing to the improvement of the quality of health care, which requires evaluation for determining its effect on the incidence of medication errors.

To our knowledge, no previous randomized controlled trials exist that were designed to evaluate the effect of a clinical pharmacy practice model (CPPM) on the incidence of medication errors (patient safety), and thus, most of these strategies are based on expert recommendations and descriptive studies [8, 28]. In Colombia, the first and only related RCT, Effectiveness of a Method for Pharmaceutical Care in outpatients with Bipolar disorder, showed the risk of hospitalizations and emergencies was lower for the intervention group [29]. This study is the first stepped-wedge controlled trial designed to assess the effect of the clinical pharmacy practice model on the incidence of medication error in hospitalized patients. Also, this study could generate strong evidence, both theoretical and practical, that the CPPM could contribute to reducing medication errors in hospitalized patients. The stepped-wedge design was selected for this trial due to the variability of the hospitalization wards (clusters) and their complexity in the clinical and pharmacological management of the patients. In this methodology, all clusters are first placed in the control group and then move to an intervention group, conserving power, unlike parallel studies in which power changed by the increased variability in the groups [30, 31].

## Abbreviations

ADR: adverse drug reactions
CPPM: clinical pharmacy practice model
IRB: institutional review board
ITT: intention to treat
ME: medication errors
NCC MERP: National Coordinating Council of Medication Error Reporting and Prevention
PTUH: Pablo Tobón Uribe Hospital
WHO: World Health Organization
